# Acute acetate administration increases endogenous opioid levels in the human brain: A [^11^C]carfentanil molecular imaging study

**DOI:** 10.1177/0269881120965912

**Published:** 2021-01-06

**Authors:** Abhishekh H Ashok, Jim Myers, Gary Frost, Samuel Turton, Roger N Gunn, Jan Passchier, Alessandro Colasanti, Tiago Reis Marques, David Nutt, Anne Lingford-Hughes, Oliver D Howes, Eugenii A Rabiner

**Affiliations:** 1 Psychiatric Imaging Group, MRC London Institute of Medical Sciences (LMS), Imperial College London, London, UK; 2 Psychiatric Imaging Group, Institute of Clinical Sciences (ICS), Faculty of Medicine, Imperial College London, London, UK; 3 Department of Psychosis Studies, Institute of Psychiatry, Psychology and Neuroscience, King’s College London, London, UK; 4 Department of Radiology, University of Cambridge, Cambridge, UK; 5 Department of Radiology, Addenbrooke’s Hospital, Cambridge University Hospitals NHS Foundation Trust, Cambridge, UK; 6 Imperial College London, UK; 7 Institute of Psychiatry, Psychology and Neurosciences, King’s College London, London, UK; 8 Invicro, London, UK; 9 Department of Neuroscience, Brighton and Sussex Medical School, University of Sussex, Brighton, UK

**Keywords:** Acetate, PET, opioid

## Abstract

**Introduction:**

A recent study has shown that acetate administration leads to a fourfold increase in the transcription of proopiomelanocortin (POMC) mRNA in the hypothalamus. POMC is cleaved to peptides, including β-endorphin, an endogenous opioid (EO) agonist that binds preferentially to the µ-opioid receptor (MOR). We hypothesised that an acetate challenge would increase the levels of EO in the human brain. We have previously demonstrated that increased EO release in the human brain can be detected using positron emission tomography (PET) with the selective MOR radioligand [^11^C]carfentanil. We used this approach to evaluate the effects of an acute acetate challenge on EO levels in the brain of healthy human volunteers.

**Methods:**

Seven volunteers each completed a baseline [^11^C]carfentanil PET scan followed by an administration of sodium acetate before a second [^11^C]carfentanil PET scan. Dynamic PET data were acquired over 90 minutes, and corrected for attenuation, scatter and subject motion. Regional [^11^C] carfentanil *BP*_ND_ values were then calculated using the simplified reference tissue model (with the occipital grey matter as the reference region). Change in regional EO concentration was evaluated as the change in [^11^C]carfentanil *BP*_ND_ following acetate administration.

**Results:**

Following sodium acetate administration, 2.5–6.5% reductions in [^11^C]carfentanil regional *BP*_ND_ were seen, with statistical significance reached in the cerebellum, temporal lobe, orbitofrontal cortex, striatum and thalamus.

**Conclusions:**

We have demonstrated that an acute acetate challenge has the potential to increase EO release in the human brain, providing a plausible mechanism of the central effects of acetate on appetite in humans.

## Introduction

A preclinical study has shown that a single intraperitoneal administration of acetate (500 mg/kg) led to a fourfold increase in the transcription of proopiomelanocortin (POMC) mRNA in the mouse hypothalamus which was linked to central appetite regulation (Frost et al., 2014). Acetate is formed in the colon by bacterial fermentation and tissue metabolism. Up to 70% of acetate is metabolised by the liver, where it is used as an energy source and substrate for the synthesis of cholesterol and long-chain fatty acids and as a co-substrate for glutamine and glutamate synthesis (Ballard, 1972; Knowles et al., 1974). POMC is cleaved to peptides, including β-endorphin, an endogenous opioid (EO) agonist that binds preferentially to the opioid receptor (Castro and Morrison, 1997). In vivo evaluation of any acetate-induced increases in EO released in the human brain will help us to understand the mechanism that acetate may play in human appetite regulation and its mediation by opioid signalling. In addition, acetate administration may be a useful paradigm to study EO release in neuropsychiatric disorders.

Opioid signalling plays a key role in appetite regulation and the development of obesity (Bessesen and Van Gaal, 2018). Animal studies have shown that palatable food consumption releases EO (Colantuoni et al., 2001; Dum et al., 1983). Consistent with this evidence, a human positron emission tomography (PET) study reported that feeding triggers cerebral opioid release, even in the absence of subjective pleasure effects (Tuulari et al., 2017). Moreover, PET studies in subjects with obesity have shown reduced µ-opioid receptor (MOR) availability (Joutsa et al., 2018; Karlsson et al., 2015, 2016), and it has been proposed that overeating leads to overstimulation of the MOR and concomitant downregulation.

Several pharmacological and non-pharmacological stimuli have been evaluated as methods to increase EO release. Previous PET studies with [^11^C]carfentanil and [^11^C]diprenorphine have shown that autobiographical sad mood induction, social rejection and positive emotion processing tasks lead to a change in binding in relevant brain regions (Hsu et al., 2015; Koepp et al., 2009; Prossin et al., 2016). Dexamphetamine challenge has been used successfully to demonstrate differences in EO release in healthy volunteers and patients with neuropsychiatric disorders (Colasanti et al., 2012; Mick et al., 2016). Moreover, development of a method that could enable EO release by increasing POMC transcription in patients with neuropsychiatric disorders is desirable, as the current methods use dexamphetamine, which may be challenging is some populations (e.g. psychosis or addictive disorders).

Human neuroimaging studies suggest that acetate crosses the blood–brain barrier (Chambers et al., 2015; Volkow et al., 2013). However, no data exist to indicate whether acetate could induce detectable changes in mu-opioid tracer binding, indicative of EO release, in humans. In view of these data, we used a PET imaging approach to test the hypothesis that an acetate challenge will increase the levels of EO in the brain of healthy human volunteers.

## Methods

The study was approved by the West London Research Ethics Committee and the Administration of Radioactive Substances Advisory Committee, UK. Written informed consent was obtained from all the participants. Seven healthy male volunteers were recruited into the study. Participants were screened using a medical history and physical examination, and current/previous medical and mental health, as well as the history of alcohol, tobacco and other substance use, were assessed by a trained study physician using the Mini Psychiatric Interview International (MINI-5; Sheehan et al., 1998). Subjects with current or previous psychiatric disorders were excluded. Participants were also excluded if they drank more than 14 UK units of alcohol per week. Other drug use (except tobacco) was not allowed for 2 weeks prior to the study. This was confirmed on the study day by a negative urine drug screen testing (cocaine, amphetamine, THC, methadone, opioids and benzodiazepines) and alcohol breath test. All subjects were asked to refrain from consuming caffeine and smoking on the day of the scan. None of the subjects met the criteria for any substance-use disorder.

All volunteers completed the [^11^C]carfentanil baseline PET scan in the morning followed by an infusion of 150 mmol of sodium acetate in 1 L normal saline over 60 minutes completed 30–60 minutes before a second [^11^C]carfentanil PET scan. All subjects had light food (sandwich) after the first scan. There was at least an hour’s rest period between food intake and initiation of acetate infusion. Due to technical reasons such as a delay in the production or quality-control checks, the PET tracer was injected between 20 and 70 minutes after acetate infusion. Five subjects underwent both PET scans on the same day. For two subjects, the post-acetate scan was acquired on a different day for logistical reasons.

### Data acquisition

Dynamic [^11^C]carfentanil PET scans were acquired on a HiRez Biograph 6 PET/computed tomography (CT) scanner (Siemens Healthcare, Erlangen, Germany), and data were collected continuously for 90 minutes (26 frames: 8×15 seconds, 3×60 seconds, 5×120 seconds, 5×300 seconds, 5×600 seconds), following an intravenous injection of [^11^C]carfentanil (Ashok et al., 2019). All participants underwent a T1-weighted structural magnetic resonance imaging (MRI) scan (Magnetom Trio Syngo MR B13 Siemens 3T; Siemens AG, Medical Solutions). All the structural images were reviewed by an experienced neuroradiologist for unexpected findings of clinical significance, and none were identified.

### Image analysis

The preprocessing of images and PET modelling were carried out using MIAKAT software (Gunn et al., 2016). There was no significant difference in the head motion between pre and post scan. Regional time–activity data were sampled using the CIC neuroanatomical atlas after frame-by-frame motion correction of the dynamic PET data, (Tziortzi et al., 2011). This was applied to the PET image by non-linear deformation parameters derived from the transformation of the structural MRI into standard space. Previous functional MRI and preclinical studies have shown that food cue and consumption activates the hypothalamus, frontal lobe, amygdala, orbitofrontal cortex, striatum, temporal lobe, thalamus (Devoto et al., 2018; Volkow et al., 2012, 2017), insula (Wright et al., 2016), cingulate cortex (Meng et al., 2018) and cerebellum (Zhu and Wang, 2008), and these areas have a high density of MOR and can be reliably quantified. Based on these data, 10 grey-matter-masked regions of interest were chosen a priori based on the work above and the evidence of sufficient density of MOR that can be reliably quantified by PET imaging in the human brain (Colasanti et al., 2012). The simplified reference tissue model (Lammertsma and Hume, 1996) with the occipital lobe as the reference region (Colasanti et al., 2012) was used to derive regional *BP*_ND_ values at each PET scan. EO release was indexed as the fractional reduction in [^11^C]carfentanil *BP*_ND_ following the sodium acetate challenge:

$$\Delta BP_{\mathrm{ND}}=\frac{\left(BP_{\mathrm{NDpre}}-BP_{\mathrm{NDpost}}\right)}{BP_{\mathrm{NDpre}}}$$.

Demographic, radiochemical and binding potential parameters were analysed using paired *t*-tests (two-tailed), and values are expressed as the mean±standard deviation. All statistical comparisons were assessed using IBM SPSS Statistics for Windows v20.0 (IBM Corp., Armonk, NY). *p*-Values of <0.05 were accepted statistically significant.

## Results

**Figure 1.**
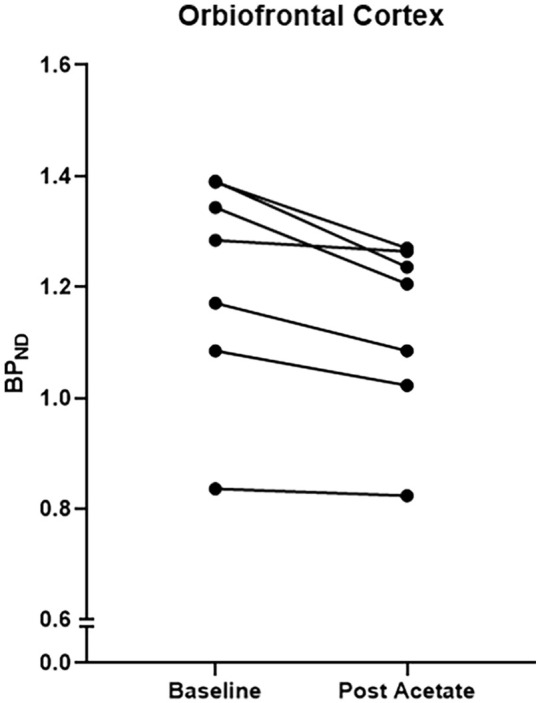
Change in [^11^C]carfentanil binding potential in the orbitofrontal cortex following administration of sodium acetate administration.

The mean age of the group was 39.1±10.5 years, and the body mass index was 24±3 kg/m^2^. There was no significant difference in the injected mass (Pre-acetate vs. Post acetate: 1.93±0.33 vs. 1.84±0.26 µg; *p*=0.53) and injected activity (Pre-acetate vs. Post acetate: 210±26.9 vs. 240±43 MBq; *p*=0.1). Following sodium acetate administration, [^11^C]carfentanil *BP*_ND_ was reduced in all regions and was statistically significant in the cerebellum, temporal lobe, orbitofrontal cortex, striatum and thalamus (*p*<0.05; Table 1; Figure 1). Repeated-measures analysis of variance showed a significant effect of acetate administration on [^11^C]carfentanil *BP*_ND_ measures (*p*<0.05). Participants did not report a subjective difference in appetite following acetate infusion. Our cerebellar and orbitofrontal cortex findings survived Benjamini–Hochberg correction (*p*<0.05).

**Table 1.** *BP*_ND_ at baseline and after acetate administration.

Brain region	Baseline *BP*_ND_, *M* (*SD*)	Post acetate *BP*_ND_, *M* (*SD*)	∆*BP_ND_* (%)	*p*-Value
Cerebellum	0.82 (0.18)	0.78 (0.18)	5.1	0.003*
Insular cortex	1.53 (0.18)	1.47 (0.23)	3.9	0.110
Temporal lobe	1.03 (0.13)	0.99 (0.11)	4.3	0.020*
Frontal lobe	0.85 (0.15)	0.82 (0.13)	3.0	0.190
Cingulate cortex	1.29 (0.21)	1.26 (0.21)	2.7	0.110
Amygdala	1.62 (0.5)	1.66 (0.16)	6.9	0.860
Orbitofrontal cortex	1.21 (0.2)	1.12 (0.16)	6.5	0.007*
Striatum	1.83 (0.17)	1.73 (0.15)	5.3	0.020*
Thalamus	1.66 (0.09)	1.57 (0.12)	5.0	0.030*
Hypothalamus	1.82 (0.24)	1.75 (0.29)	3.0	0.510

* *p*<0.05.

*SD*: standard deviation.

## Discussion

We have shown reductions in the binding of a MOR selective radiotracer – [^11^C]carfentanil – that are consistent with an increase in EO release in the human brain following acetate administration. Our data are consistent with our hypothesis that an acetate challenge will increase the levels of EO in the brain of healthy human volunteers. The change in [^11^C]carfentanil binding reached statistical significance in the orbitofrontal cortex, striatum, thalamus, cerebellum and the temporal lobe.

A previous study in mice showed that acetate derived from the fermentation of carbohydrate in the colon alters POMC neuron activity (Frost et al., 2014). We have now shown that peripheral acetate administration alters opioid signalling in the human brain. We did not detect a significant change in EO in the hypothalamus, where acetate administration was shown to alter POMC expression and neuronal activity in the mouse, and the location of POMC-expressing neurons (Frost et al., 2014). We measured opioid release rather than POMC expression or neuronal activity, and EO release may be expected in regions anatomically distant from the neuronal cell bodies in the hypothalamus. If acetate stimulates the production of EO peptides in hypothalamic neurons, these would be transported via axonal projections, and EO released in the striatum and cortical regions involved in food salience, where stimulation of MOR may occur (Cameron et al., 2017).

The mechanism of acetate-induced appetite regulation is speculative at this stage. The available evidence suggests that acetate enters the astrocytes, where it is metabolised in the TCA cycle, resulting in an increase in malonyl-CoA. The studies have also demonstrated that acetate increases the glutamate–glutamine cycle, triggering Ca2+ uptake. It remains unclear whether the changes in POMC mRNA expression (Frost et al., 2014) are causally related to the cellular changes above. The POMC neurons are heterogeneous, with varying levels of receptor expression (Toda et al., 2017). It has been suggested that melanocortin–opioid interactions are crucial in the regulation of feeding behaviour, as they are secreted in the same vesicle (Millington, 2007). Our study further supports the hypothesis that peripheral short-chain fatty acids such as acetate regulate central neurochemical signalling in the key regions involved in appetite regulation.

Our results have implications in understanding the association between acetate and its effect on brain regions implicated in the rewarding effect of alcohol. In a state of alcohol intoxication, the concentration of acetate in the blood is reported to increase to 0.5–1 mM (Korri et al., 1985; Orrego et al., 1988). Alcohol infusion has been shown to decreases brain glucose metabolism and increase [^11^C] acetate utilisation (Volkow et al., 2013). Consistent with this study, our results show elevated EO levels in the brain regions reported to have increased acetate utilisation in the intoxicated state. In addition, acute alcohol ingestion is shown to release EO (Mitchell et al., 2012), and there is blunting of opioid release in abstinent alcohol-dependent individuals (Turton et al., 2018). Together, these studies show that acetate-induced opioid release may be involved in the hedonic response to alcohol and alcohol-induced appetite suppression.

Our study has certain limitations. First, this is a small sample size pilot study, and our findings should be replicated in a larger number of subjects. Although we saw a reduction in the *BP*_ND_ values across all the brain regions, statistical significance was not seen in the insula, frontal lobe, cingulate cortex, amygdala and hypothalamus. Moreover, the magnitude of the change in [^11^C]carfentanil *BP*_ND_ PET signal is modest and may not be sufficient for robust quantification of dose-dependent effects. Second, we did not measure acetate plasma levels post administration. A previous PET study reported good brain uptake of [^11^C]acetate administered through an intravenous route (Volkow et al., 2013). The measurement of plasma acetate would have been useful. However, we felt that within the confines of a pilot study this was not essential, as our primary aim was to detect whether a large acetate load produced any effects on the brain EO system at all. If we were to find such an effect, subsequent investigations would evaluate the nature of a dose–effect relationship between acetate dose and magnitude of EO response. We believe that within the limited number of subjects in our study and the limited dose range used, measurement of plasma acetate would mainly serve the purpose of identifying subjects who have not achieved a meaningful increase in plasma acetate concentration due to experimental variability. As we administered sodium acetate by the intravenous route, we believe that the likelihood of substantial differences in plasma acetate exposure between subjects is low, and hence we omitted this measurement for practical and logistic reasons. The dose of acetate was chosen on the basis of safety, by evaluating the literature and by determining the highest reported dose of acetate administered previously. A consistent elevation of acetate plasma concentration to approximately 1.4 mmol/L was reported following the administration of 150 mmol of sodium acetate (Akanji et al., 1990).

Second, the preclinical study suggested that POMC transcription increased 30 minutes after acetate infusion. In our study, due to logistical reasons, subjects completed infusion 20–70 minutes before the scan, and the scan was acquired over 90 minutes. The time course of increases in EO following an elevation of POMC mRNA in the human brain is a matter of conjecture, and we may have missed the peak endogenous release in some or all of our subjects. Future microdialysis studies are needed to explore the b-endorphin concentration changes (Maidment et al., 1989) over time, following acetate administration. Third, order effect is a confounding factor, as our study was not counterbalanced. Previous studies have indicated that placebo may lead to alterations in the opioid system (Pecina and Zubieta, 2015; Pecina et al., 2015). Follow-up studies with larger sample sizes and controlling for variables such as order effects, diurnal variation and the potential for placebo responses will be required to test conclusively the hypothesis that acute acetate administration leads to enhanced EO levels in the human brain.

Finally, previous studies have reported [^11^C]carfentanil *BP*_ND_ test–retest variability of 10% (Hirvonen et al., 2009). The magnitude of change noted in our study is lower than this threshold. However, our results are consistent across brain regions in all study subjects.

We have demonstrated that an acute acetate challenge has the potential to increase EO release in the human brain, providing a plausible mechanism of the central effects of acetate on appetite in humans.
